# Serum ischemia-modified albumin in patients with type 2 diabetes mellitus with and without diabetic nephropathy: A cross-sectional study

**DOI:** 10.3892/mi.2026.346

**Published:** 2026-10-23

**Authors:** Bharat Ramrao Sontakke, Vishwajit Deshmukh, Bharat Singh Rathod, Jancy Jose, Satyendra Tripathi, Yash Bisht, Ashok Kumar Ahirwar

**Affiliations:** 1 Department of Anatomy, All India Institute of Medical Sciences (AIIMS), MIHAN, Dahegaon, Maharashtra 441108, India; 2 Department of Medicine, All India Institute of Medical Sciences (AIIMS), MIHAN, Dahegaon, Maharashtra 441108, India; 3 Department of Biochemistry, All India Institute of Medical Sciences (AIIMS), MIHAN, Dahegaon, Maharashtra 441108, India; 4 Department of Laboratory Medicine, All India Institute of Medical Sciences (AIIMS), New Delhi, Delhi 110029, India

**Keywords:** ischemia-modified albumin, diabetic nephropathy, type 2 diabetes mellitus, oxidative stress, chronic kidney disease

## Abstract

Diabetic nephropathy is the leading cause of end-stage renal disease worldwide, and ischemia-modified albumin (IMA) has been proposed as a biomarker of oxidative stress in type 2 diabetes mellitus (T2DM). The present hospital-based cross-sectional study evaluated serum IMA levels together with biochemical, haematological, urinary and comorbidity profiles in patients with T2DM with and without nephropathy. The primary outcome was the between-group difference in serum IMA; there was no healthy control group. A total of 50 patients were enrolled between November, 2021 and March, 2024, including 25 patients with T2DM without nephropathy (group A) and 25 patients with diabetic nephropathy (group B). Clinical and laboratory records were available for 19 patients in group A and 16 patients in group B; the others contributed IMA values only. In group B, chronic kidney disease (CKD) stage 5D was documented in 9 patients, CKD on maintenance haemodialysis in 1 patient, acute-on-chronic kidney disease in 1 patient, CKD of unspecified stage in 4 patients, and 10 patients had no clinical record. Serum IMA levels did not differ significantly between the groups [group A: Median, 0.332 absorbance units (ABSU); group B: Median, 0.317 ABSU; Mann-Whitney test; P=0.727; mean difference (group A vs. B), 0.054 ABSU, 95% confidence interval, -0.08 to 0.19]; thus, a clinically relevant difference cannot be excluded. The result was similar following adjustment for serum albumin and in sensitivity analyses (all P>0.5). Blood urea (P=0.0003) and serum creatinine (P<0.0001) levels were higher in group B; the serum potassium level was nominally higher (P=0.040), but within the normal range in both groups. Among the patients with records, anaemia (14/18, 78% vs. 10/15, 67%) and infection (12/18, 67% vs. 9/15, 60%) were more frequent in group A than in group B. Heavy proteinuria (+++/++++) was recorded only in group B (2/7 tested) and all adverse in-hospital outcomes occurred in group B; however, these comparisons rest on very small numbers. In the present small study without a healthy control group, serum IMA did not differ between patients with T2DM with and without nephropathy, and the findings are compatible with IMA reflecting systemic oxidative stress rather than nephropathy-specific injury. Larger prospective studies with standardized IMA assays, albumin adjustment and comprehensive biochemical profiling are required to clarify the clinical utility of IMA in diabetic kidney disease.

## Introduction

Diabetes mellitus is a chronic metabolic disorder, of worldwide significance, with >537 million adults living with diabetes as of 2021, projected to increase to almost 783 million by the year 2045 (1,2). In India, this disease burden is more prominent; the country is home to >77 million individuals afflicted by diabetes, rendering India a leader internationally in terms of diabetes prevalence (3). Diabetic nephropathy (DN) is the most common microvascular complication of type 2 diabetes and a leading cause of end-stage renal disease globally, and is a major contributor to co-morbidity, mortality and health-care costs (4-8). In spite of the advancements made in management, the early and consistent identification of biomarkers for the onset and progression of nephropathy remain elusive in routine practice.

Ischemia-modified albumin (IMA) is an altered version of human serum albumin which has been structurally modified at the N-terminal cobalt binding site due to either hypoxia, ischaemia, or free radicals (9). IMA was originally validated as a marker of myocardial ischaemia, but has recently gained significant attention as an indicator of systemic oxidative stress associated with type 2 diabetes mellitus (T2DM) (9,10). In patients with diabetes, chronic hyperglycaemia leads to the excessive production of reactive oxygen species (ROS) via numerous pathways, including the generation of advanced glycation end products, the activation of the polyol pathway and the stimulation of protein kinase C (11), resulting in the structural alteration of albumin and thus, increased levels of IMA (12). Albumin from patients with diabetes exhibits an enhanced oxidative modification and impaired copper-binding capacity (13). Previous studies have compared the levels of IMA in subjects with T2DM with those in healthy controls and demonstrated an increase in IMA in patients with diabetes (12,14,15), although lower levels were reported in one study (16) and higher levels have also been reported in prediabetes (17); however, some studies have suggested that increased levels of IMA are also associated with patients with DN (15,18-20) and, more generally, with kidney disease (21,22). The variation in the data across studies and assays, as well as disease state underscores the necessity for more methodologically rigorous comparisons of IMA levels in diverse patient populations with type 2 diabetes. A key feature of the albumin cobalt binding (ACB) assay is that its result depends on the amount of albumin in the sample; hypoalbuminaemia, which is common in diabetic kidney disease, can therefore influence the apparent IMA value, independently of ischaemia or oxidative stress.

The present study thus compared serum IMA levels between patients with T2DM with and without DN in a single cohort from a tertiary care academic centre in central India, and examined its associations with biochemical, haematological, urinary and clinical parameters. It was hypothesised that serum IMA levels would be higher in patients with nephropathy than in those without. The objectives specified in the approved study protocol were the following: i) To compare serum IMA levels between patients with T2DM with and without nephropathy, which was the primary objective [primary outcome: The between-group difference in serum IMA, in absorbance units (ABSU)]; ii) To measure haematological and biochemical parameters in the study subjects; iii) to examine the correlation of these parameters with serum IMA; and iv) to examine comorbidities in the study subjects. The analysis reported herein covers the complete blood count (CBC), kidney function, liver function and HbA1c data available in the records; urinalysis findings and in-hospital outcomes are also described.

## Patients and methods

### Study design

The present study was a hospital-based cross-sectional observational study conducted at the Departments of Biochemistry and Medicine of the All India Institute of Medical Sciences (AIIMS), Nagpur, a tertiary care hospital in India. Participants were recruited between November, 2021 and March, 2024. The present study is reported in accordance with the STROBE guideline for cross-sectional studies. The present study was reviewed and approved by the Institutional Ethics Committee (IEC; IEC/Pharmac/2020/190; approval date, January 9, 2021). The study was conducted in accordance with the ethical principles of the Declaration of Helsinki. Written informed consent was obtained from all participants prior to inclusion in the study.

### Study groups and sampling

A total of 50 patients were divided into two groups as follows: Group A (n=25), which included patients with T2DM without DN; and group B (n=25), which included patients with T2DM with established DN. Participants were enrolled using lottery-based random sampling from the T2DM inpatient and outpatient pool; inpatients and outpatients were both eligible, and the number from each setting is reported in Table I. Nephropathy was defined by the spot urinary albumin-creatinine ratio (UACR) of ≥30 mg/g creatinine and/or an estimated glomerular filtration rate (eGFR) <60 ml/min/1.73m², confirmed on at least two occasions, in the absence of other primary renal pathology (23). eGFR was calculated with the CKD-EPI 2021 creatinine equation (24). The eGFR values, the UACR values that were available in the laboratory reports and the haemodialysis status are reported in Table I.

### Inclusion and exclusion criteria

All patients with established T2DM with a duration of at least 5 years were eligible. The exclusion criteria included the following: Active chronic inflammatory conditions (tuberculosis on treatment, rheumatoid arthritis, systemic lupus erythematosus, inflammatory bowel disease, connective tissue disorders) or other debilitating non-diabetic illnesses likely to confound IMA. Acute infection and anaemia were not considered as exclusion criteria, as the aim of the present study was to investigate a representative hospital-based population of patients with T2DM, in whom these conditions are common. Both are recognised confounders of IMA, as they increase hypoxia and oxidative stress, and they were therefore recorded and examined in stratified and adjusted analyses, as described below. Of note, one haemolysed sample in each group was retained in the primary analysis; a sensitivity analysis excluding both is reported below.

### Investigations

All blood samples were collected following an overnight fast. Blood samples were centrifuged at 3,000 rpm (1,006 x g) for 10 min at room temperature to separate serum. Fresh samples were analysed, and the remaining samples were stored at -80˚C. Investigations included the following:

i) Serum IMA by ACB spectrophotometric assay (9,25), performed as previously described by the authors (26). Briefly, 200 µl serum were mixed with 50 µl of 0.1% cobalt chloride (Sigma-Aldrich; Merck KGaA) and, after gentle mixing, left for 10 min for cobalt-albumin binding; 50 µl dithiothreitol (DTT; Sigma-Aldrich; Merck KGaA; 1.5 mg/ml in distilled water) was then added, and the reaction was terminated 2 min later with 1 ml of 0.9% sodium chloride (Thermo Fisher Scientific, Inc.). The colour developed was read at 470 nm against a serum blank without DTT on a spectrophotometer (UV Plus, Motras Scientific Instruments Pvt. Ltd.) and expressed in ABSU. Serum IMA was also adjusted for serum albumin (IMA/albumin ratio in ABSU per g/dl, and the analysis of covariance (ANCOVA) with albumin as the covariate).

ii) CBC: Haemoglobin, haematocrit, red blood cells (RBC), white blood cells (WBC), platelet count, mean corpuscular haemoglobin (MCH), mean corpuscular haemoglobin concentration (MCHC), mean corpuscular volume and red cell distribution width-coefficient of variation (RDW-CV).

iii) Kidney function tests: Blood urea, serum creatinine, sodium, potassium; eGFR by the CKD-EPI 2021 creatinine equation (24) (creatinine clearance by the Cockcroft-Gault equation, which is not normalised to body surface area, was used in the original analysis).

iv) Liver function tests (LFTs): Albumin, total protein, alanine transaminase, aspartate transaminase, alkaline phosphatase and total bilirubin.

v) HbA1c by HPLC on the ADAMS A1c HA-8180V automatic glycohaemoglobin analyser (ARKRAY, Inc.).

vi) Complete urinalysis: Dipstick and microscopy.

vii) Diabetic retinopathy, assessed with the assistance of an ophthalmologist. Lipid profile data were unavailable and are acknowledged as a limitation of the present study.

### Statistical analysis

All data were analysed using IBM SPSS Statistics v20.0. The normality of all continuous variables was assessed using the Shapiro-Wilk (SW) test. Normally distributed variables are presented as the mean ± SD and compared using an independent-samples Student's t-test. Non-normally distributed variables are presented as the median and interquartile range (IQR) and compared using the Mann-Whitney U (MWU) test. Since serum IMA was non-normally distributed in group A (SW, P=0.015), Spearman's rank correlation coefficient (ρ) was used for all IMA correlation analyses in both groups. Qualitative urine parameters were compared using Fisher's exact test. The primary comparison (serum IMA, group A vs. group B) is reported with the mean difference and its 95% confidence interval (CI) and with effect sizes (Hedges' g; rank-biserial correlation r = Z/√N for the Mann-Whitney test); effect sizes and 95% CIs for the other comparisons are presented in Table SI. CIs for Spearman's ρ were obtained by Fisher z-transformation (Bonett-Wright standard error), and exact (Clopper-Pearson) 95% CIs are provided for proportions. The sample size of 25 per group was calculated at the protocol stage, assuming mean IMA values of 81 and 90 units (SD 10 units) in the two groups, a type I error of 5 and 80% power, which yielded 25 patients per group; these assumed values are expressed on a different scale from the ABSU used in the present study; thus, the precision of the primary comparison is also described: With 25 patients per group, the study had 80% power (two-sided α=0.05) to detect only a difference of ~0.19 ABSU (standardised difference, 0.81), which is 3.5-fold the observed difference. Serum IMA was additionally analysed following adjustment for serum albumin (IMA/albumin ratio and ANCOVA), and a multivariable linear regression model for serum IMA included group, serum albumin, haemoglobin, dialysis status and infection. Sensitivity analyses excluded patients documented to have received haemodialysis and excluded the two haemolysed samples; serum IMA was also compared within strata of anaemia, infection and (in group B) haemodialysis. All patients with clinical records were inpatients; thus, an inpatient-outpatient comparison was not possible. Clinical and laboratory records were available for 19 of 25 patients in group A and for 16 of 25 patients in group B; the remaining patients (6 and 9, respectively) contributed IMA values only. Missing data were not imputed, analyses are complete-case, and the number of observations for each variable is stated in the tables. Of note, ~60 statistical tests were performed. No correction for multiple testing was applied, all analyses other than the primary comparison are exploratory, and for 60 tests a Bonferroni-adjusted significance threshold would be P<0.0008. Otherwise, a P-value <0.05 was considered to indicate a nominally statistically significant difference.

## Results

### Participant characteristics and group classification

Baseline characteristics are presented in Table I. Serum IMA was measured in all 50 patients; clinical and laboratory records were available for 19 of 25 patients in group A and 16 of 25 patients in group B (comorbidity data for 18 and 15 patients), all of whom were hospital inpatients, while the remaining 6 and 9 patients, respectively, contributed IMA values only. The mean age was similar (55.7±13.2 vs. 56.2±12.4 years; P=0.898), although a larger proportion of patients in group B were men (10/16, 63% vs. 5/19, 26%; Fisher's test, P=0.044). A UACR value was documented in the laboratory reports of 5 patients in group A and 3 patients in group B (Table I). By the CKD-EPI 2021 equation, eGFR was <60 ml/min/1.73 m² in all 16 patients in group B with a creatinine result (median, 7.8; IQR, 5.5-12.9) and in 7 of 19 patients in group A (median, 73.1; IQR, 54.1-102.8). Group assignment was made at enrolment and was retained for all analyses. The 2 patients in group A with markedly increase urea and creatinine levels (urea up to 264 mg/dl, creatinine up to 5.2 mg/dl; Fig. 1B) had documented Acute-on-chronic kidney disease. According to the clinical records, 11 of the 16 patients in group B with records received haemodialysis (maintenance haemodialysis or haemodialysis that commenced during the admission), and 2 of the 19 patients in group A received haemodialysis for acute kidney injury. The CKD stage distribution in group B was as follows: CKD stage 5D, 9 patients (36%); CKD on maintenance haemodialysis, 1 patient (4%); AKI on CKD, 1 patient (4%); CKD of unspecified stage, 4 patients (16%); and no clinical record available, 10 patients (40%).

### Serum IMA, renal function and glycaemic parameters

The principal finding was that serum IMA levels did not differ significantly between the groups [group A median, 0.332 ABSU (IQR, 0.173-0.464; mean, 0.352±0.268); group B median, 0.317 ABSU (IQR, 0.087-0.455; mean, 0.297±0.203); MWU P=0.727]. The mean difference (group A - group B) was 0.054 ABSU (95% CI, -0.08 to 0.19; Hedges' g 0.22, 95% CI, -0.32 to 0.77; rank-biserial r +0.06). The data are therefore compatible with a difference ranging from ~0.08 ABSU lower to 0.19 ABSU higher in group A, and a clinically relevant difference cannot be excluded. Blood urea levels were significantly higher in group B (median, 97.8 vs. 29.3 mg/dl; MWU P=0.0003), as were serum creatinine levels (median, 7.12 vs. 1.00 mg/dl; MWU P<0.0001); both remained significant following the Bonferroni correction for ~60 tests (threshold P<0.0008). The serum potassium level was nominally higher in group B (4.38±0.61 vs. 3.93±0.63 mEq/l; t-test P=0.040); however, both group mean values were within the normal range and the difference does not survive correction for multiple testing. HbA1c and serum sodium did not differ significantly. Serum IMA, renal function and glycaemic parameters are summarized in Table II. The distribution of serum IMA, blood urea and serum creatinine is illustrated in Fig. 1.

### Liver function and haematological parameters

All LFT and CBC parameters were comparable between the groups (Table III). The mean haemoglobin level was in the anaemic range in both groups (group A, 9.69 g/dl; group B, 9.08 g/dl; P=0.329) and the mean serum albumin level was low in both groups (3.08 and 3.10 g/dl; P=0.936). The WBC count was higher in group B, although the difference was not statistically significant (11.53±5.41 vs. 8.68±3.00 x10³/µl; P=0.063). RDW-CV was higher in group A, although the difference was not statistically significant (median 15.8 vs. 14.7%; P=0.071). The number of observations varied by variable (CBC, n=18 in group A and n=14-16 in group B; Table III), as CBC data were available for only 18 of 25 patients in group A and 14-16 of 25 patients in group B (the others had IMA values only).

### Spearman's correlations of serum IMA

Since serum IMA was non-normally distributed in group A (SW, P=0.015), Spearman's rank correlation was applied for all IMA correlation analyses in both groups. In group A, the only nominally significant correlation was a negative correlation between IMA and MCHC (ρ=-0.502; 95% CI, -0.80 to -0.02; P=0.034, n=18; Fig. 2). This analysis is exploratory: It is one of ~60 uncorrected tests, it does not reach the Bonferroni-adjusted threshold (P<0.0008), and the number of 18 patients (rather than 25 patients) reflects the availability of a complete blood count for 18 of the 25 patients in group A. The association depends on two extreme observations (IMA, 0.02 ABSU with MCHC 35.1 g/dl; and IMA, 1.07 ABSU with MCHC 32.1 g/dl); after excluding these, ρ was -0.37 (95% CI, -0.74 to 0.17; P=0.157; n=16) (Fig. 2). It is therefore regarded as hypothesis-generating only. Other correlations did not reach statistical significance, including IMA vs. MCH (ρ=-0.454, P=0.058) and IMA vs. RBC count (ρ=0.441, P=0.067) in group A, and IMA vs. RDW-CV (ρ=0.468, P=0.091) in group B. In group B, IMA did not correlate with serum creatinine (ρ=0.074) or blood urea (ρ=-0.071); that is, IMA did not vary with the degree of renal impairment within the nephropathy group. The complete correlation data are presented in Table IV.

### Urinalysis, comorbidities and adverse outcomes

Among the patients with a comorbidity record, Diabetic retinopathy was recorded in 6/15 (40%) patients in group B and 1/18 (6%) patients in group A (Fisher's test, P=0.030, nominal and uncorrected for multiple testing), and CKD stage 5D in 9/15 (60%) of patients in group B with records (Fig. 3). Anaemia (14/18, 78% vs. 10/15, 67%; Fisher's test, P=0.697) and infectious conditions (12/18, 67% vs. 9/15, 60%; Fisher's test, P=0.731) were more frequent in group A than in group B; both are recognised confounders that would be expected to raise IMA in group A relative to group B. Complete urine data were available for 17/25 patients in group A and 7-10/25 patients in group B (denominators for individual findings are presented in Table V). Urinalysis parameters were not available for all participants as the corresponding urine investigation reports were unavailable in the clinical records at the time of data extraction. Overall proteinuria prevalence did not differ significantly (18% vs. 43%; Fisher's test, P=0.307). Heavy proteinuria (+++ or ++++) was recorded in 2/7 tested patients in group B (29%; exact 95% CI, 4-71%) and in 0/17 patients in group A (0%; 95% CI, 0-20%) (Fisher's test, P=0.077; Fig. 4). As only 7 patients in group B were tested, this comparison is very imprecise and should be interpreted with caution. Pus cells (40-53%) and microscopic haematuria (29-47%) were common in both groups. All adverse outcomes [one in-hospital mortality, two left against medical advice (LAMA) and one dialysis refusal] occurred in group B. The full data are presented in Table V.

### Sensitivity and adjusted analyses of serum IMA

The serum albumin level was low in both groups (3.08±0.70 and 3.10±0.41 g/dl). Following adjustment for albumin, IMA still did not differ between the groups (IMA/albumin ratio: Group A median, 0.120; group B, 0.094 ABSU per g/dl; P=0.512; ANCOVA-adjusted difference; group A - Group B, 0.055 ABSU; 95% CI, -0.12 to 0.23, P=0.526). In a multivariable linear regression model including group, serum albumin, haemoglobin, haemodialysis and infection (29 patients with complete data), the adjusted difference between groups (group A - group B) was 0.01 ABSU (95% CI, -0.24 to 0.26; P=0.93). The between-group comparison was similar after excluding patients documented to have received haemodialysis (group A, 23; group B, 14; difference, 0.043 ABSU; 95% CI, -0.13 to 0.22; P=0.913) and after excluding the two haemolysed samples (P=0.749) (Table VI). Within group B, IMA did not differ between patients who received haemodialysis and those who did not (median, 0.263 vs. 0.386 ABSU; P=0.583). Among patients with records, IMA did not differ between those with and without anaemia (median, 0.329 vs. 0.274 ABSU; P=0.424) or with and without infection (0.332 vs. 0.289 ABSU; P=0.749). These analyses are small and imprecise, and all confidence intervals remain wide. All patients with records were inpatients; thus, a separate inpatient-outpatient comparison was not possible.

## Discussion

### IMA and DN: Primary finding in context

The principal finding of the present study was that serum IMA did not differ significantly between patients with T2DM with and without nephropathy in this clinically complex hospital-based population (mean difference, 0.054 ABSU; 95% CI, -0.08 to 0.19). The CI is compatible with no difference, but also with differences in either direction that could be clinically relevant; thus, the result is inconclusive rather than evidence of equivalence; and, as there was no healthy control group, the study cannot demonstrate that the assay discriminates diabetes or disease from health. One interpretation, which was examined in post hoc analyses (as demonstrated above), is that IMA reflects systemic oxidative burden rather than nephropathy-specific injury. The result was similar whether analysed parametrically (t-test P=0.425) or non-parametrically (P=0.727), although agreement between statistical tests does not address confounding or sample size. This finding is in contrast to previous reports of significantly higher IMA in nephropathic than in non-nephropathic T2DM groups by Ukinc *et al* (15), Hussein (18) and Ahmed *et al* (19). The difference reported by Ukinc *et al* (15) (0.355 vs. 0.311 ABSU) is of similar magnitude to, but opposite in direction from, the difference observed herein (0.054 ABSU); however, the dispersion they reported (±0.035 and ±0.046 ABSU) is several-fold smaller than the standard deviations in the present study cohort (0.20-0.27 ABSU), consistent with greater heterogeneity of the hospital-based sample in the present study.

The discordance between studies may be explained, at least in part, by IMA biologically being a marker of systemic oxidative burden rather than nephropathy-specific injury. IMA can be increased by any condition generating ROS or tissue hypoxia, including hypertension, ischaemic heart disease, anaemia and severe infections, such as sepsis (27), all of which were common in both groups (group A vs. group B, among patients with records: 56% vs. 67%, 17% vs. 20%, 78% vs. 67% and 67% vs. 60%, respectively); anaemia and infection were in fact more frequent in group A. Comorbidity-associated oxidative stress may therefore have narrowed any difference in IMA between the groups. The present study examined this explanation directly: IMA did not differ significantly between patients with and without anaemia (median, 0.329 vs. 0.274 ABSU; P=0.42) or with and without infection (0.332 vs. 0.289 ABSU; P=0.75), although these analyses were small and imprecise (as demonstrated above). As the present study was a cross-sectional study, these observations do not establish that IMA elevation in T2DM is driven by comorbidity rather than by loss of glomerular filtration.

A further consideration is the uremic milieu of patients in group B. Several patients had severely elevated creatinine levels (median, 7.12 mg/dl), 9 (36%) patients had CKD stage 5D, and 11 of the 16 patients with records received haemodialysis. IMA has been reported to be raised in end-stage renal disease (21) and in CKD and patients undergoing haemodialysis, with the glomerular filtration rate the main determinant of IMA in one cohort (28); by contrast, IMA did not vary with creatinine within the nephropathy group herein (ρ=0.074). The reasons for this are uncertain; they may include the small sample size, the heterogeneity of comorbid conditions and the effects of dialysis, which were examined only by excluding patients who received haemodialysis (Table VI).

### Renal function markers and clinical stratification

The marked elevation in blood urea (MWU P=0.0003) and serum creatinine (MWU P<0.0001) levels in group B is consistent with the clinical classification of this group. The median creatinine level of 7.12 mg/dl in group B, with 36% having CKD stage 5D, indicates an advanced nephropathy group. The mean serum potassium level was slightly higher in group B (4.38 vs. 3.93 mEq/l; P=0.040), a difference that does not survive correction for multiple testing; both means are within the normal range, and these data do not indicate hyperkalaemia.

No statistically significant differences in liver function tests were detected between the groups; however, with 25 patients per group this does not demonstrate that potential confounders were balanced.

### Urinalysis findings and proteinuria burden

Heavy proteinuria (+++ / ++++) was recorded only in group B (2/7 tested, 29%; 95% CI, 4-71%, vs. 0/17 in group A; Fisher's test, P=0.077). The difference was not statistically significant and rests on small number of patients; however, its direction is consistent with the macroalbuminuria characteristic of established DN (29,30). The high prevalence of pus cells and microscopic haematuria in both groups (40-53 and 29-47%, respectively) may be attributable to the heavy concurrent infectious burden, predominantly urinary tract infection (UTI), sepsis and pneumonia, rather than to primary glomerular pathology. Afkarian *et al* (31) noted that the risk of developing a UTI was substantially elevated in T2DM due to glycosuria-mediated bacterial growth and impaired neutrophil function, consistent with the infectious profile observed herein.

### Comorbidities, microvascular co-progression and adverse outcomes

The higher prevalence of diabetic retinopathy in group B (6/15, 40% vs. 1/18, 6%; Fisher's test, P=0.030, nominal; small numbers) is in keeping with the well-recognised co-progression of diabetic microvascular complications. Zheng *et al* (32) documented that the incidence of retinopathy closely parallels nephropathy progression, sharing common upstream pathophysiology of chronic hyperglycaemia-induced microvascular endothelial dysfunction. The 2022 KDIGO guidelines (6) and the 2024 ADA Standards of Care (1) both recommend simultaneous ophthalmological and renal screening in T2DM. Thomas *et al* (33) outlined the shared molecular mechanisms, including podocyte injury, mesangial expansion and tubular hypoxia, linking glomerular and retinal microvascular disease in diabetes.

All adverse outcomes, one in-hospital mortality (4%), two LAMA events (8%) and one dialysis refusal (4%), were confined exclusively to group B, which may reflect both the clinical severity of advanced-stage CKD and the socioeconomic and attitudinal barriers to dialysis acceptance in India. Jha *et al* (2013) (34) highlighted that dialysis acceptance rates in South Asia are significantly constrained by financial burden, logistic barriers and cultural attitudes toward end-stage treatment. Tancredi *et al* (35) demonstrated that patients with T2DM with renal impairment carry substantially higher excess mortality, a pattern mirrored in the outcomes of group B in the present study cohort, although four events are too minimal for firm conclusions. Cardiovascular comorbidity was common in both groups (hypertension, 56 and 67%; ischemic heart disease/coronary artery disease, 17 and 20%).

### IMA as a uremic oxidative stress marker: Broader implications

IMA is not currently recommended for nephropathy detection in KDOQI 2012(5) or KDIGO 2022(6) guidance, and standardisation of the ACB assay across laboratories is lacking. The present study adds a small and imprecise negative dataset, without a healthy control group, to this evidence base.

The present study has certain critical limitations which should be mentioned: i) There was no healthy control group; thus, the ability of the assay to discriminate diabetes or disease from health was not demonstrated in this cohort; ii) the sample size was small (25 per group); the 95% CI for the primary comparison (-0.08 to 0.19 ABSU) does not exclude a clinically relevant difference, and the study could reliably detect only differences of ~0.19 ABSU; iii) the ACB assay is albumin-dependent and hypoalbuminaemia was present in both groups (mean, 3.08 and 3.10 g/dl); albumin adjustment was performed post hoc; iv) anaemia and acute infection, both recognised to increase IMA, were common in both groups; v) 9 (36%) patients in group B had CKD stage 5D, and uraemia and dialysis may themselves alter IMA; vi) urine data were missing for up to 72% of patients in group B, which limits the urinalysis comparisons; vii) multivariable adjustment was limited by the sample size; viii) ~60 comparisons were made without correction for multiplicity; thus, isolated nominally significant findings (for example IMA-MCHC and potassium) may be chance findings; ix) the cross-sectional design precludes inference on causality or on the temporal association between IMA and nephropathy; x) ABSU values depend on the assay protocol and are not comparable across laboratories; xi) single-centre recruitment at a tertiary referral hospital may have introduced referral bias; xii) clinical and laboratory records were available for only 19 of 25 patients in group A and 16 of 25 patients in group B (the others contributed IMA values only); thus, secondary analyses had as few as five observations (HbA1c in group B); all patients with records were inpatients, and one haemolysed sample per group was retained in the primary analysis. The findings presented herein warrant validation in larger prospective cohorts with healthy controls, standardised assays, albumin adjustment, complete biochemical profiling and UACR-based nephropathy staging.

In conclusion, in the present study, serum IMA did not significantly differentiate patients with T2DM with and without DN (MWU P=0.727; mean difference, 0.054 ABSU; 95% CI, -0.08 to 0.19); however, the confidence interval does not exclude a clinically relevant difference and the study had no healthy control group. The findings are compatible with IMA reflecting systemic oxidative burden rather than nephropathy-specific injury in clinically complex populations; this requires confirmation however. Renal function markers (creatinine, urea) confirmed group stratification and the severity of the clinical profile in group B, including CKD stage 5D in 36% and all adverse outcomes, indicates a clinically advanced nephropathy group. An inverse IMA-MCHC association in group A (Spearman ρ=-0.502, Pp=0.034) is exploratory and hypothesis-generating only, given the small sample, its dependence on two extreme observations, and the absence of multiple comparison correction. Serum IMA should be regarded as a candidate marker of systemic oxidative stress rather than a nephropathy-staging tool, pending validation in larger prospective cohorts with healthy controls, albumin adjustment and standardised assays.

## Supplementary Material

Between-group comparison of serum IMA and clinical laboratory parameters in patients with type 2 diabetes mellitus without (group A) and with (group B) diabetic nephropathy.

## Figures and Tables

**Figure 1:**
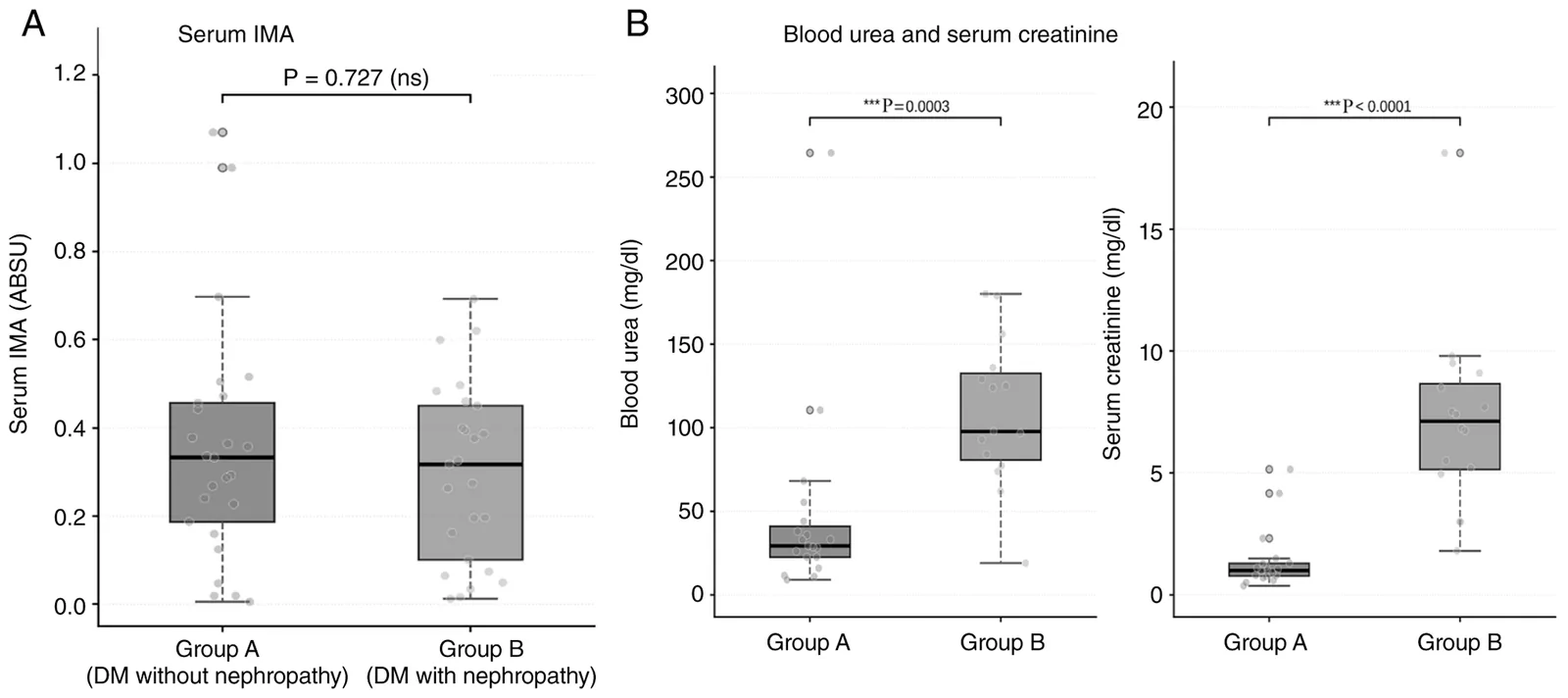
Serum IMA and kidney function test parameters. (A) Serum IMA (ABSU) in group A vs. group B; MWU P=0.727 (ns). (B) Blood urea (MWU ^***^P=0.0003) and serum creatinine (MWU ^***^P<0.0001) were significantly elevated in group B. Boxes represent the IQR; lines represent the median; dots represent individual patients. Several patients in group A had markedly increased urea or creatinine levels (as described above in the Results). IMA, ischemia-modified albumin; ABSU, absorbance units; MWU, Mann-Whitney U test; IQR, interquartile range; ns, not significant; DM, diabetes mellitus.

**Figure 2:**
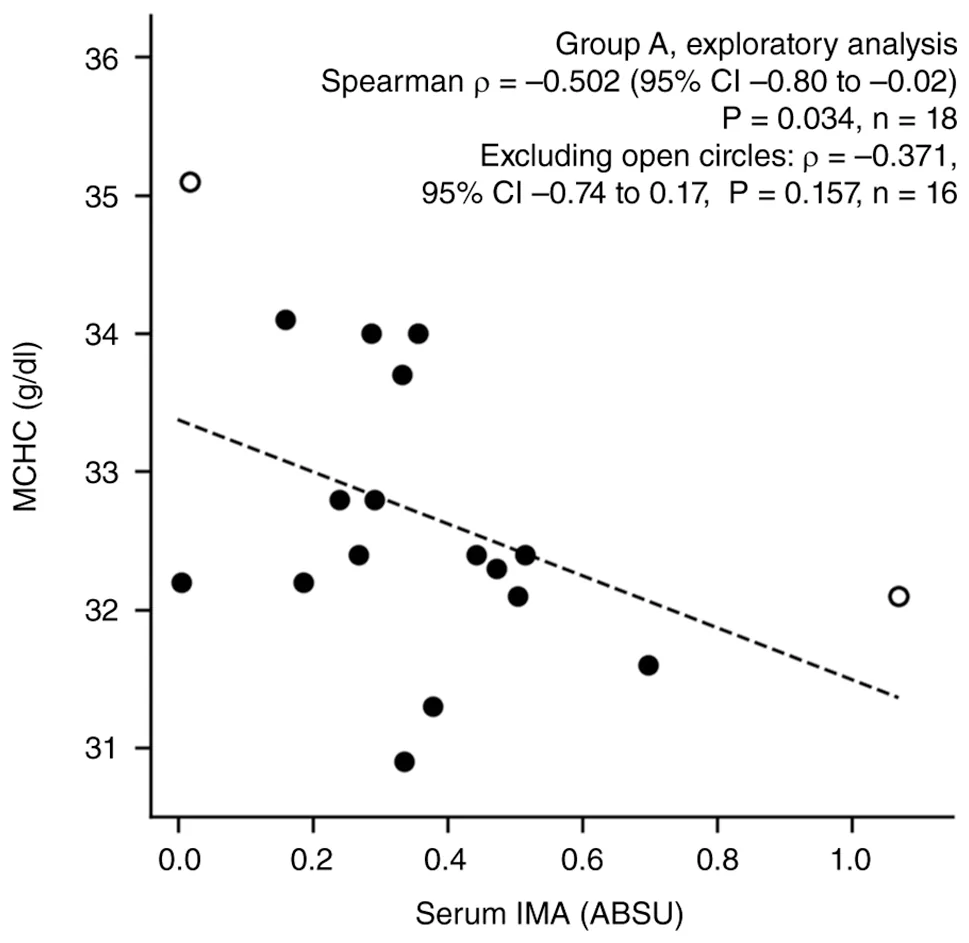
Spearman's rank correlation analysis between serum IMA (ABSU) and MCHC (g/dl) in group A (exploratory analysis). ρ=-0.502 (95% CI, -0.80 to -0.02), P=0.034, n=18; after excluding the two extreme observations (open circles), ρ=-0.371 (95% CI, -0.74 to 0.17), P=0.157, n=16. The dashed line represents the regression line for visual reference only. IMA, ischemia-modified albumin; ABSU, absorbance units; MCHC, mean corpuscular haemoglobin concentration.

**Figure 3:**
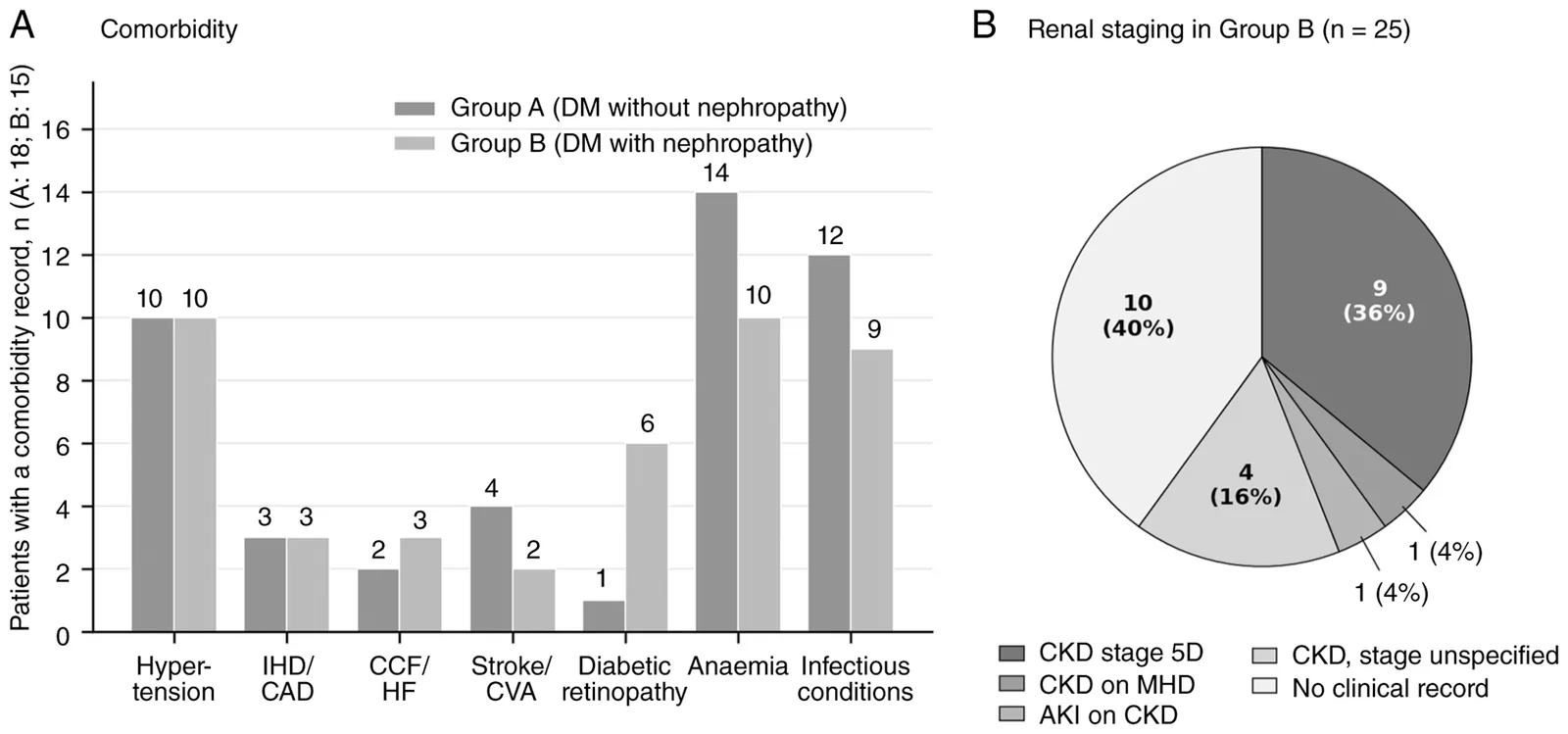
Comorbidity and renal staging profiles. (A) Comorbidity distribution in group A vs. group B (number of patients with a comorbidity record: Group A, n=18; group B, n=15). Diabetic retinopathy was recorded in 6/15 patients in group B and 1/18 patients in group A. (B) Renal staging in group B (n=25; 10 patients had no clinical record available): CKD stage 5D 9 (36%), CKD on maintenance haemodialysis 1 (4%), AKI on CKD 1 (4%), CKD of unspecified stage 4 (16%), no clinical record available 10 (40%). CKD, chronic kidney disease; AKI on CKD, acute-on-chronic kidney disease; MHD, maintenance haemodialysis; IHD, ischemic heart disease; CAD, coronary artery disease; CCF, congestive heart failure; HF, heart failure; CVA, cerebrovascular accident.

**Figure 4:**
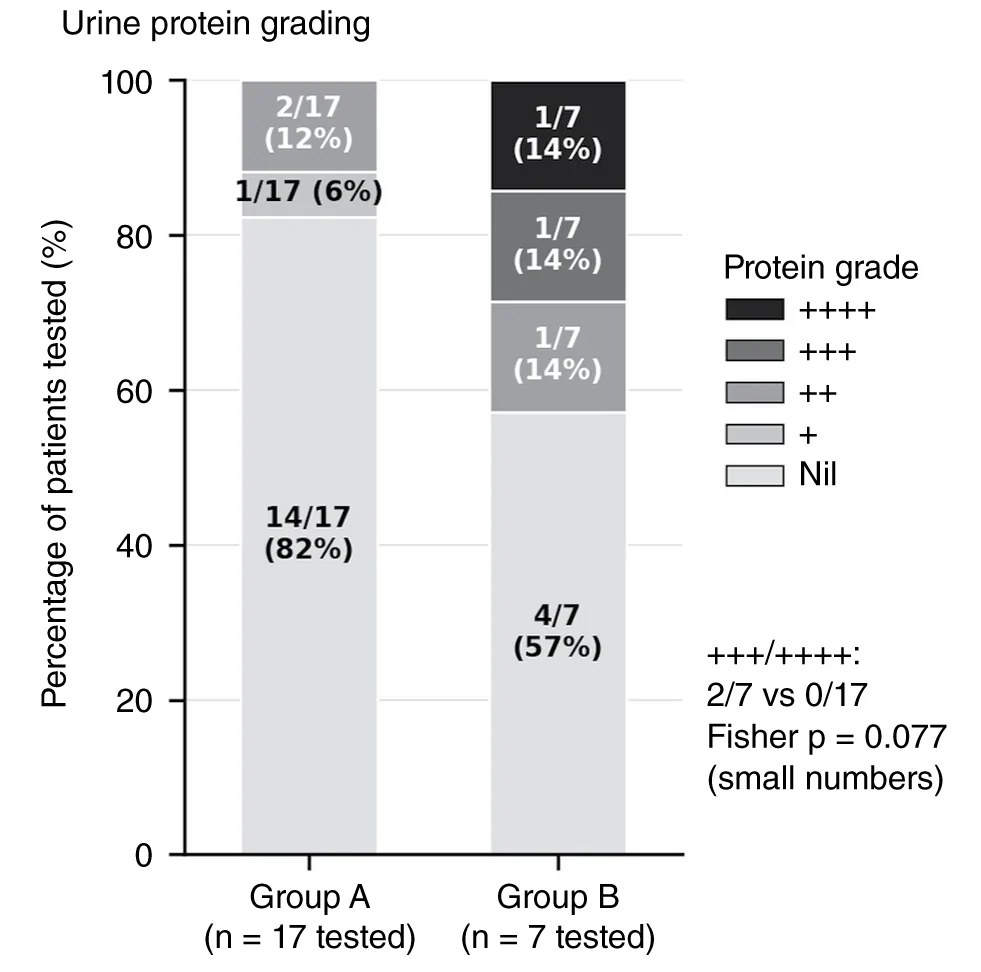
Urine protein grading. Dipstick protein grade in patients with urine data (group A, n=17; group B, n=7). Heavy proteinuria (+++/++++) was recorded in 2/7 patients in group B and 0/17 patients in group A (Fisher's test, P=0.077); the numbers are very minimal and the comparison is imprecise.

**Table I:** Baseline characteristics and group classification (group A vs. group B).

Characteristic	Group A (n=25)	Group B (n=25)	P-value
Patients with IMA measured, n	25	25	-
Patients with clinical/laboratory records, n	19	16	-
Age (years), mean ± SD	55.7±13.2 (n=19)	56.2±12.4 (n=16)	0.898
Male sex, n/n (%)	5/19 (26%)	10/16 (63%)	0.044
Setting (patients with records)	Inpatients 19/19	Inpatients 16/16	-
Systolic BP at discharge (mmHg), median (IQR) (n)	125 (110-140) (11)	136 (126-158) (5]	-
Diastolic BP at discharge (mmHg), median (IQR) (n)	72 (64-80) (11)	90 (80-100) (5)	-
Insulin documented, n/n	10/19	6/16	-
Oral anti-diabetic agents documented, n/n	10/19	7/16	-
RAAS inhibitors documented, n/n	1/19	0/16	-
Statins documented, n/n	6/19	4/16	-
Received haemodialysis (clinical records), n/n	2/19 (for AKI)	11/16 (69%)	-
UACR (mg/g), documented in laboratory reports: n; median (range)	5; 106.7 (<13-1652)	3; 4029.8 (333.3-12720)	-
eGFR, CKD-EPI 2021 (ml/min/1.73 m²), median (IQR)	73.1 (54.1-102.8) (n=19)	7.8 (5.5-12.9) (n=16)	-
eGFR <60 ml/min/1.73 m², n/n (%)	7/19 (37%)	16/16 (100%)	-
eGFR <15 ml/min/1.73 m², n/n (%)	2/19 (11%)	13/16 (81%)	-

Values are presented as the mean ± SD, median (IQR or range) or n/n (%). Records include patients with laboratory and clinical data in the hospital record (group A, n=19; group B, n=16); the remaining 6 and 9 patients, respectively contributed IMA values only. Blood pressure, medications and haemodialysis were obtained from the discharge summaries (treatment administered, discharge advice and hospital course); a medication that was not documented was not assumed to be unused. UACR values are the first values listed in the laboratory reports where available. Original Cockcroft-Gault values are not shown. Group A, patients with diabetes mellitus without nephropathy; Group B, patients with diabetes mellitus with nephropathy; IMA, ischemia-modified albumin; BP, blood pressure; RAAS, renin-angiotensin-aldosterone system; UACR, urinary albumin-creatinine ratio; eGFR, estimated glomerular filtration rate.

**Table II:** Serum IMA, renal function, and glycaemic parameters (group A vs. group B).

Parameter	n (A/B)	Group A	Group B	Test	P-value	Effect size/difference
Serum IMA (ABSU)	25/25	0.332 (0.173-0.464)	0.317 (0.087-0.455)	MWU	0.727 (ns)	MD (A-B) 0.054 (95% CI, -0.08 to 0.19); g=0.22; r=+0.06
IMA/albumin ratio (ABSU per g/dl)	17/14	0.120 (0.075-0.155)	0.094 (0.045-0.151)	MWU	0.512 (ns)	MD (A-B) 0.024 (-0.04 to 0.08)
Blood urea (mg/dl)	19/15	29.3 (22.6-44.0)	97.8 (77.3-136.0)	MWU	0.0003^a^	r=-0.74
Serum creatinine (mg/dl)	19/16	1.00 (0.76-1.30)	7.12 (5.01-8.95)	MWU	<0.0001^a^	r=-0.95
Serum potassium (mEq/l)	19/15	3.93±0.63	4.38±0.61	t-test	0.040 (nominal)	MD -0.46 (-0.89 to -0.02)
Serum sodium (mEq/l)	19/14	135.0±4.6	133.0±5.0	t-test	0.259 (ns)	MD 1.9 (-1.5 to 5.4)
HbA1c (%)	14/5	7.2 (6.8-9.7)	8.1 (6.4-8.2)	MWU	0.610 (ns)	r=-0.17

Data are presented as the median (IQR) for non-normal variables and as the mean ± SD for normal variables.

^a^Remains significant following the Bonferroni correction for ~60 tests (P<0.0008); the potassium difference does not. IMA non-normal in Group A (Shapiro-Wilk test, P=0.015). For HbA1c, group A values include 18.1, 13.7 and 12.3% (verified against the laboratory report for 18.1%); Group B, n=5. MWU, Mann-Whitney U test; IQR, interquartile range; MD, mean difference (group A vs. group B); g, Hedges' g; r, rank-biserial correlation (positive=higher in group A); n (A/B), number of observations in group A/group B (patients without a result for a variable are not counted); IMA, ischemia-modified albumin; ABSU, absorbance units.

**Table III:** Liver function test and complete blood count parameters (group A vs. group B).

A, Liver function tests						
Parameter	n (A/B)	Group A	Group B	Test	P-value	Effect size/difference
Albumin (g/dl)	17/14	3.08±0.70	3.10±0.41	t-test	0.936 (ns)	MD -0.02 (-0.45 to 0.41)
Total protein (g/dl)	17/14	5.96 (5.05-6.80)	6.32 (5.68-6.71)	MWU	0.302 (ns)	r=-0.22
ALT (U/l)	17/14	17.0 (13.0-28.6)	17.5 (12.9-23.0)	MWU	0.781 (ns)	r=-0.06
AST (U/l)	17/14	26.4 (18.0-43.9)	22.9 (18.8-34.2)	MWU	0.766 (ns)	r=+0.07
Alk. phosphatase (U/l)	17/14	93.0 (71.0-111.5)	112.0 (83.2-198.8)	MWU	0.104 (ns)	r=-0.35
Total bilirubin (mg/dl)	17/13	0.47 (0.32-0.98)	0.48 (0.39-0.94)	MWU	0.586 (ns)	r=-0.12
B, Complete blood count						
Parameter	n (A/B)	Group A	Group B	Test	P-value	Effect size/difference
Haemoglobin (g/dl)	18/15	9.69±1.89	9.08±1.57	t-test	0.329 (ns)	MD 0.61 (-0.64 to 1.86)
Haematocrit (%)	18/14	29.63±5.78	27.67±4.79	t-test	0.314 (ns)	MD 1.96 (-1.95 to 5.87)
RBC (x10^6^/µl)	18/14	3.27 (3.08-4.26)	3.39 (2.83-4.06)	MWU	0.894 (ns)	r=+0.03
WBC (x10³/µl)	18/16	8.68±3.00	11.53±5.41	t-test	0.063 (ns)	MD -2.85 (-5.86 to 0.16)
Platelet count (x10³/µl)	18/15	227 (180-323)	245 (179-295)	MWU	0.759 (ns)	r=-0.07
MCH (pg)	18/14	26.7 (23.5-28.5)	26.8 (25.2-27.8)	MWU	0.909 (ns)	r=-0.03
MCHC (g/dl)	18/14	32.69±1.09	33.04±1.00	t-test	0.352 (ns)	MD -0.35 (-1.12 to 0.41)
MCV (fl)	18/14	80.3 (72.3-87.6)	80.9 (76.9-83.6)	MWU	0.835 (ns)	r=+0.05
RDW-CV (%)	18/14	15.8 (14.2-17.6)	14.6 (13.3-15.5)	MWU	0.071 (ns)	r=+0.38

Data are presented as the median (IQR) for non-normal variables, and as the mean ± SD for normal variables. MWU or t-test was applied following Shapiro-Wilk normality testing. ns, not significant for all parameters.; n (A/B), number of observations in group A/group B; MD, mean difference (group A - group B); r, rank-biserial correlation (positive, higher in group A); MWU, Mann-Whitney U test; ALT, alanine aminotransferase; AST, aspartate aminotransferase; RBC, red blood cells; WBC, white blood cells; MCH, mean corpuscular haemoglobin; MCHC, mean corpuscular haemoglobin concentration; MCV, mean corpuscular volume; RDW-CV, red cell distribution width-coefficient of variation.

**Table IV:** Spearman's rank correlations of serum IMA (group A and group B).

	Group A (without nephropathy)			Group B (with nephropathy)		
Parameter	ρ	P-value	Sig	ρ	P-value	Sig
MCHC (g/dl)	-0.502	0.034	Nominal P<0.05^a^	-0.260	0.370	ns
MCH (pg)	-0.454	0.058	ns	-0.359	0.208	ns
RBC count	0.441	0.067	ns	-	-	-
RDW-CV (%)	-	-	-	0.468	0.091	ns
Blood urea	-0.288	0.231	ns	-0.071	0.800	ns
Serum creatinine	-0.219	0.367	ns	0.074	0.787	ns
Haemoglobin	0.197	0.433	ns	-0.057	0.840	ns
WBC	-0.292	0.240	ns	0.409	0.116	ns
HbA1c	0.086	0.771	ns	-	-	-
ALT/AST	-0.281	0.274	ns	0.282	0.329	ns
Alk. phosphatase	-0.381	0.132	ns	-	-	-
Total bilirubin	-0.042	0.872	ns	-	-	-
Platelet count	-0.412	0.090	ns	-	-	-
Total protein	-	-	-	0.385	0.175	ns
Serum sodium	-	-	-	0.305	0.288	ns
MCV	-0.389	0.111	ns	-0.231	0.427	ns

Spearman's ρ used throughout as IMA was non-normally distributed (SW P=0.015 in group A). Number of observations: Group A, n=14-19 (n=18 for MCHC); group B, n=5-16 (n=14 for MCHC). No formal multiple comparison correction applied; all correlations are exploratory.

^a^Nominal P<0.05; does not meet the Bonferroni-adjusted threshold for ~60 tests (P<0.0008). ns, not significant; -, not assessed or insufficient data. MCH, mean corpuscular haemoglobin; MCHC, mean corpuscular haemoglobin concentration; RBC, red blood cell; WBC, white blood cell; ALT, alanine aminotransferase; AST, aspartate aminotransferase; MCV, mean corpuscular volume; RDW-CV, red cell distribution width-coefficient of variation.

**Table V:** Urinalysis findings, comorbidity profile and adverse outcomes (group A vs. group B).

A, Urinalysis findings (group A, n=17; group B, n=7-10 documented)				
Parameter/comorbidity	Group A n/n (%; 95% CI)	Group B n/n (%; 95% CI)	Statistical test	P-value/note
Proteinuria (any +)	3/17 (18%; 4-43)	3/7 (43%; 10-82)	Fisher's	0.307 (ns)
Heavy proteinuria (+++/++++)	0/17 (0%; 0-20)	2/7 (29%; 4-71)	Fisher's	0.077 (ns)
Glucosuria	4/17 (24%; 7-50)	2/9 (22%; 3-60)	Fisher's	ns
Haematuria (dipstick)	5/17 (29%; 10-56)	3/8 (38%; 9-76)	Fisher's	ns
Pus cells (microscopy)	9/17 (53%; 28-77)	4/10 (40%; 12-74)	Fisher's	0.695 (ns)
RBC (microscopy)	8/17 (47%; 23-72)	4/10 (40%; 12-74)	Fisher's	ns
Bacteriuria	4/17 (24%; 7-50)	2/9 (22%; 3-60)	Fisher's	ns
Casts (any)	2/17 (12%; 1-36)	1/10 (10%; 0-45)	Fisher's	ns
B, Comorbidity profile (patients with a comorbidity record: Group A, n=18; group B, n=15)				
Parameter/comorbidity	Group A n/n (%; 95% CI)	Group B n/n (%; 95% CI)	Statistical test	P-value/note
Hypertension	10/18 (56%; 31-78)	10/15 (67%; 38-88)	Fisher's	P=0.722 (ns)
IHD/CAD	3/18 (17%; 4-41)	3/15 (20%; 4-48)	Fisher's	ns
CCF/HF	2/18 (11%; 1-35)	3/15 (20%; 4-48)	Fisher's	P=0.639 (ns)
Stroke/CVA	4/18 (22%; 6-48)	2/15 (13%; 2-40)	Fisher's	P=0.665 (ns)
Diabetic retinopathy	1/18 (6%; 0-27)	6/15 (40%; 16-68)	Fisher's	P=0.030 (nominal)
Anaemia	14/18 (78%; 52-94)	10/15 (67%; 38-88)	Fisher's	P=0.697 (ns); more frequent in group A
Infectious conditions	12/18 (67%; 41-87)	9/15 (60%; 32-84)	Fisher's	P=0.731 (ns); more frequent in group A
CKD Stage 5D	-	9/15 (60%; 32-84)	-	Group B only
C, In-hospital adverse outcomes (patients with discharge records: Group A, n=19; group B, n=16)				
Parameter/comorbidity	Group A n/n (%; 95% CI)	Group B n/n (%; 95% CI)	Statistical test	P-value/note
In-hospital mortality	0/19 (0%; 0-18)	1/16 (6%; 0-30)	-	Group B only
Left against medical advice (LAMA)	0/19 (0%; 0-18)	2/16 (12%; 2-38)	-	Group B only
Refused dialysis	-	1/16 (6%; 0-30)	-	Group B only

Fisher's exact test was used for urinalysis parameters and for the comorbidities shown. Values are presented as n/n (%; exact Clopper-Pearson 95% CI). Urinalysis parameters were not available for all participants as the corresponding urine investigation reports were unavailable in the clinical records at the time of data extraction (group A, n=17; group B, n=7-10); comparisons in group B rest on very small numbers and should be interpreted with caution. Comorbidity denominators are the numbers of patients with a comorbidity record (group A, n=18; group B, n=15). ns, not significant; RBC, red blood cells; IHD, ischemic heart disease; CAD, coronary artery disease; CCF, congestive heart failure; HF, heart failure; CVA, cerebrovascular accident; CKD, chronic kidney disease.

**Table VI:** Sensitivity and adjusted analyses of serum IMA.

Analysis	n (A/B)	IMA, group A or stratum 1: median (IQR)	IMA, group B or stratum 2: median (IQR)	Difference (95% CI)	P-value
Primary analysis: all patients	25/25	0.332 (0.173-0.464)	0.317 (0.087-0.455)	0.054 (-0.08 to 0.19)	0.727
Albumin-adjusted IMA (IMA/albumin ratio, ABSU per g/dl)	17/14	0.120 (0.075-0.155)	0.094 (0.045-0.151)	0.024 (-0.04 to 0.08)	0.512
ANCOVA adjusted for albumin (group A vs. group B)	17/14	-	-	0.055 (-0.12 to 0.23)	0.526
Multivariable model (group, albumin, haemoglobin, haemodialysis, infection) (group A vs. group B)	16/13	-	-	0.011 (-0.24 to 0.26)	0.930
Excluding patients documented to have received haemodialysis	23/14	0.336 (0.187-0.472)	0.347 (0.084-0.486)	0.043 (-0.13 to 0.22)	0.913
Excluding the two haemolysed samples	24/24	0.312 (0.166-0.453)	0.295 (0.081-0.437)	0.053 (-0.08 to 0.19)	0.749
Group B only: haemodialysis (stratum 1) vs. none (stratum 2)	11/5	0.263 (0.074-0.399)	0.386 (0.145-0.548)	-0.082	0.583
Patients with records: anaemia (stratum 1) vs. no anaemia (stratum 2)	24/11	0.329 (0.189-0.432)	0.274 (0.019-0.472)	0.088	0.424
Patients with records: infection (stratum 1) vs. no infection (stratum 2)	21/14	0.332 (0.173-0.461)	0.289 (0.140-0.423)	0.064	0.749

All analyses are exploratory and uncorrected for multiple testing. Mann-Whitney P-values (ANCOVA and regression P-values for the adjusted models). Group A vs. group B for group comparisons; stratum 1 vs. stratum 2 for strata. The multivariable model included 29 patients with complete data (16 patients in group A and 13 patients in group B). All patients with records were inpatients. MD, mean difference; IMA, ischemia-modified albumin; ABSU, absorbance units; IQR, interquartile range.

## Data Availability

The data generated in the present study may be requested from the corresponding author.
