# Supplementary material for: Serum ischemia-modified albumin in patients with type 2 diabetes mellitus with and without diabetic nephropathy: A cross-sectional study
Source: Med Int (Lond). 2026 Oct 23;6(6):62. doi: 10.3892/mi.2026.346 (PMC13728762; doi:10.3892/mi.2026.346)
Supplement: Supplementary file 1 [file Supplementary_Data.pdf]

Table SI. Between-group comparison of serum IMA and clinical laboratory parameters in patients with type 2 diabetes mellitus without (group A) and with (group B) diabetic nephropathy.

Parameter	No. of patients		Group A					Group B					P-value		Mean difference (group A vs. group B)			Effect size		Hodges-Lehmann shift (group A vs. group B), bootstrap 95% CI	
	Group A	Group B	Median	Q1	Q3	Mean	SD	Median	Q1	Q3	Mean	SD	MWU	t-test	Diff	CI low	CI high	Hedges g	Rank-biserial r	Low	High
Serum IMA (ABSU)	25	25	0.332	0.173	0.464	0.352	0.268	0.317	0.087	0.455	0.297	0.203	0.7269	0.425	0.054	-0.081	0.189	0.22	0.06	-0.095	0.169
Blood urea (mg/dl)	19	15	29.3	22.6	44	46.689	57.742	97.8	77.3	136	108.867	44.317	0.0003	0.0016	−62.177	-98.969	-25.386	-1.16	-0.74	-100.2	-46
Serum creatinine (mg/dl)	19	16	1	0.76	1.3	1.392	1.237	7.12	5.012	8.95	7.165	3.775	<0.0001	<0.0001	-5.773	-7.64	-3.907	-2.09	-0.95	-7.13	-3.995
Potassium (mEq/l)	19	15	3.96	3.55	4.47	3.926	0.626	4.4	4.03	4.8	4.382	0.605	0.052	0.0402	-0.456	-0.89	-0.022	-0.72	-0.4	-0.95	-0.04
Sodium (mEq/l)	19	14	135	133.2	138.2	134.974	4.607	134.05	129.75	137.05	133.036	5.031	0.3624	0.2594	1.938	-1.502	5.378	0.39	0.19	-1.5	5.05
HbA1c (%)	14	5	7.2	6.775	9.675	8.614	3.619	8.1	6.45	8.2	7.48	1.341	0.6103	0.5094	1.134	-2.417	4.685	0.34	-0.17	-1.15	2.2
Albumin (g/dl)	17	14	2.9	2.64	3.62	3.083	0.697	3.07	2.758	3.4	3.1	0.408	0.6055	0.9362	-0.017	-0.449	0.415	-0.03	-0.11	-0.45	0.29
Total protein (g/dl)	17	14	5.96	5.05	6.8	5.984	1.106	6.32	5.678	6.712	6.251	0.803	0.3018	0.4578	-0.267	-0.991	0.458	-0.26	-0.22	-1.05	0.25
ALT (U/l)	17	14	17	13	28.6	24.282	20.15	17.5	12.9	23.025	25.564	27.313	0.7809	0.8816	-1.282	-18.725	16.161	-0.05	-0.06	-6	5.4
AST (U/l)	17	14	26.4	18	43.85	31.594	17.844	22.95	18.75	34.25	30.543	23.378	0.7658	0.888	1.051	-14.088	16.191	0.05	0.07	-6.6	16
Alk phosphatase (U/l)	17	14	93	71	111.5	118.588	102.212	112	83.25	198.75	162.286	117.991	0.1035	0.2782	-43.697	-124.572	37.178	-0.39	−0.35	-94	3
Total bilirubin (mg/dl)	17	13	0.47	0.315	0.98	0.842	1.015	0.48	0.39	0.94	0.972	1.437	0.5862	0.7726	-0.131	-1.047	0.786	-0.1	-0.12	-0.25	0.3
Haemoglobin (g/dl)	18	15	9.65	8.15	11.275	9.689	1.89	8.9	8.1	9.8	9.08	1.574	0.4054	0.3285	0.609	-0.642	1.86	0.34	0.17	-0.7	1.9

Haematocrit (%)	18	14	28.95	24.575	34.775	29.633	5.784	28.1	23.95	30.425	27.671	4.788	0.3925	0.3139	1.962	-1.95	5.874	0.36	0.18	-2.101	6.3
RBC (x10^6/ul)	18	14	3.275	3.08	4.255	4.317	3.123	3.39	2.832	4.062	3.456	0.662	0.8942	0.32	0.862	-0.878	2.601	0.35	0.03	-0.37	0.76
WBC (x10^3/ul)	18	16	8.495	6.328	10.348	8.677	3.002	10.625	7.905	13.965	11.529	5.41	0.0649	0.0625	−2.853	-5.864	0.158	-0.65	-0.38	-5.31	-0.15
Platelets (x10^3/ul)	18	15	227	180	323	260.167	135.523	245	179	295	243.733	91.724	0.7586	0.6926	16.433	-67.544	100.411	0.14	-0.07	-69.525	67
MCH (pg)	18	14	26.7	23.475	28.5	26.706	4.543	26.75	25.175	27.825	26.571	1.711	0.9092	0.9174	0.134	-2.486	2.755	0.04	-0.03	-2.3	1.4
MCHC (g/dl)	18	14	32.4	32.1	33.775	32.689	1.089	33.25	32.325	33.65	33.043	0.996	0.2615	0.3517	-0.354	-1.118	0.41	-0.33	-0.24	-1.2	0.5
MCV (fl)	18	14	80.35	72.325	87.625	81.567	12.004	80.9	76.875	83.575	80.45	4.497	0.8345	0.744	1.117	-5.804	8.037	0.11	0.05	-5.301	5.6
RDW-CV (%)	18	14	15.8	14.25	17.55	17.1	4.668	14.65	13.275	15.475	14.621	1.372	0.0709	0.0648	2.479	-0.162	5.119	0.67	0.38	0	2.901

Descriptive statistics, effect sizes and 95% confidence intervals are presented. All analyses other than the primary comparison (serum IMA) are exploratory and uncorrected for multiple testing. Group A, patients with type 2 diabetes mellitus without diabetic nephropathy; group B, patients with type 2 diabetes mellitus with diabetic nephropathy; n, number of patients with a result for the variable; Q1 and Q3, first and third quartiles; SD, standard deviation; MWU, Mann-Whitney U test; t-test, independent-samples Student's t-test; Diff, mean difference (group A vs. group B) with its 95% CI (low, high); Hedges g, standardised mean difference; rank-biserial r is positive when values are higher in group A; Hodges-Lehmann shift, median shift (group A vs. group B) with bootstrap 95% CI; IMA, ischaemia modified albumin; ABSU, absorbance units; ALT, alanine aminotransferase; AST, aspartate aminotransferase; ; RBC, red blood cell; WBC, white blood cell; MCH, mean corpuscular haemoglobin; MCHC, mean corpuscular haemoglobin concentration; MCV, mean corpuscular volume; RDW-CV, red cell distribution width-coefficient of variation.
